# Deduction of Novel Genes Potentially Involved in the Effects of Very Low Dose Atropine (0.003%) Treatment on Corneal Epithelial Cells Using Next-Generation Sequencing and Bioinformatics Approaches

**DOI:** 10.3390/medicina55090589

**Published:** 2019-09-13

**Authors:** Wei-An Chang, Yu-Ting Hsiao, Hsien-Chung Lin, Shu-Fang Jian, Yi-Jen Chen, Po-Lin Kuo

**Affiliations:** 1 Graduate Institute of Clinical Medicine, College of Medicine, Kaohsiung Medical University, Kaohsiung 807, Taiwan; 2 School of Medicine, College of Medicine, Kaohsiung Medical University, Kaohsiung 807, Taiwan; 3 Division of Pulmonary and Critical Care Medicine, Kaohsiung Medical University Hospital, Kaohsiung 807, Taiwan; 4 Department of Medical Education, Kaohsiung Chang Gung Memorial Hospital, Kaohsiung 833, Taiwan; 5 Department of Ophthalmology, Kaohsiung Chang Gung Memorial Hospital, Kaohsiung 833, Taiwan; 6 Department of Ophthalmology, Kaohsiung Medical University Hospital, Kaohsiung 807, Taiwan; 7 Department of Physical Medicine and Rehabilitation, Kaohsiung Medical University Hospital, Kaohsiung 807, Taiwan; 8 Center for Cancer Research, Kaohsiung Medical University, Kaohsiung 807, Taiwan

**Keywords:** corneal epithelial cells, atropine, apoptosis, next-generation sequencing, bioinformatics

## Abstract

*Background and Objectives:* Atropine is a nonselective muscarinic antagonist which has been used to prevent worsening of myopia in children. Different concentrations of atropine were used for myopia, ranging from 0.01% to 1.0%. However, there are still potential toxicity of different doses of atropine to the cornea. Here, we present a study of investigating novel genes potentially involved in the effects of very low dose atropine treatment (0.003%) on corneal epithelial cells using next-generation sequencing (NGS) and bioinformatics approaches. *Materials and Methods:* Human corneal epithelial cells were treated with 0.003% atropine, cultured until confluence, and RNA extracted for differential expression profiling of mRNA and microRNA (miRNA) between control and atropine-treated corneal epithelial cells. The functional enrichment analysis for differentially expressed genes was performed using two bioinformatics databases, including Database for Annotation, Visualization and Integrated Discovery (DAVID) and Ingenuity^®^ Pathway Analysis (IPA). In addition, potential miRNA-mRNA interactions involved in atropine-treated corneal epithelial cells were predicted and validated using different miRNA target prediction databases. *Results:* Our results showed 0.003% atropine might suppress the apoptosis of corneal epithelial cells, potentially through Ras and protein kinase A signaling pathways. We also validated the possible miRNA regulations by using TargetScan and miRDB databases. Hsa-miR-651-3p-*EPHA7*, hsa-miR-3148-*TMEM108* and hsa-miR-874-5p-*TBX6* were validated as possible miRNA regulations involved in corneal epithelial cells treated with 0.003% atropine. *Conclusions:* These findings may contribute novel insights into therapeutic strategies for treating cornea with 0.003% atropine.

## 1. Introduction

Atropine is a nonselective muscarinic antagonist which has been used to prevent worsening of myopia in children. Different concentrations of atropine were used for myopia (low dose: 0.01%, moderate dose: 0.01%~0.5% and high dose: 0.5%~1.0%) [1]. However, the potential cytotoxic effect of topical atropine treatment to the cornea is still a major concern. In a previous study, atropine was proved to have dose-dependent cytotoxicity to human corneal epithelial cells above the concentration of 0.03% by inducing mitochondrion-dependent apoptosis [2]. In a multicenter study, higher incidence rate of side effects was observed in 1% atropine eye drops groups than 0.5% atropine and 0.25% atropine eye drops groups, with symptoms of flush and fever reported [3]. In a study using low dose atropine on school-aged children, the results showed low dose (0.01%) atropine could slow myopia progression while significantly less side effects were observed when compared to higher concentration treatment [4]. Compared to moderate and high dose atropine, low dose atropine might have less side effects.

To investigate the role of very low dose atropine (0.003%) on the effect of corneal epithelial cells, next generation sequencing (NGS) was used for differential expression profiling between normal and atropine-treated corneal epithelial cells, and further functional enrichment analysis was carried out using different bioinformatics tools in the current study.

## 2. Materials and Methods

### 2.1. Study Design

**Figure 1:**
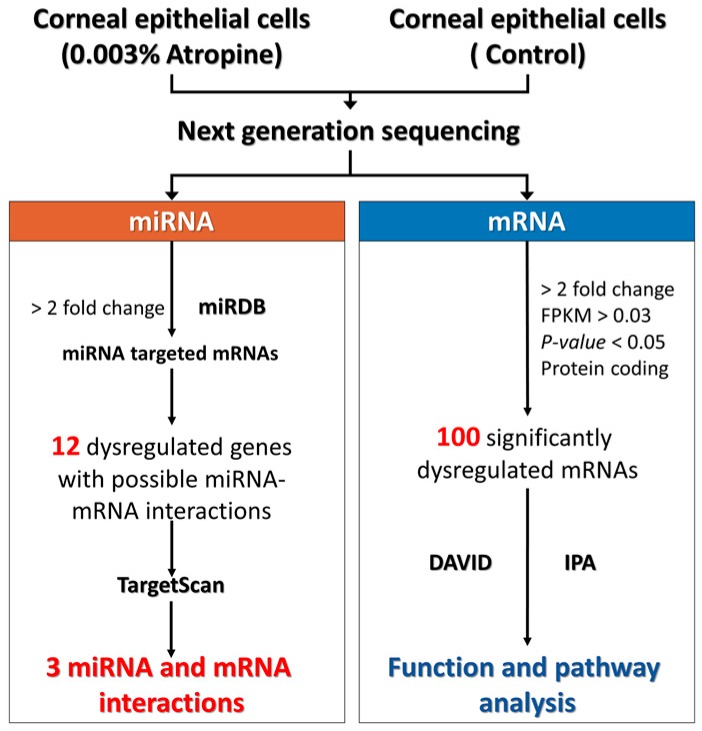
Flowchart of the study.

The flowchart of the study design is illustrated in Figure 1. Normal human corneal epithelial cells were purchased from LifeLine Technologies and cultured according to the manufacturer’s instructions in OcuLife medium (LifeLine Tech, Oceanside CA) supplemented with OcuLife growth factors (LifeLine Tech, Oceanside CA) [5]. Two bioinformatics tools were used in this study, including the Database for Annotation, Visualization and Integrated Discovery (DAVID) and Ingenuity^®^ Pathway Analysis (IPA). With these bioinformatics tools, we investigated the enriched functions and pathways related to the dysregulated genes in 0.003% atropine-treated corneal epithelial cells. For validating the potential targets of the significantly dysregulated microRNAs (miRNAs), we used TargetScan and miRDB. Then the potential miRNA-mRNA interactions involved in atropine-treated corneal epithelial cells were identified.

### 2.2. NGS for miRNA and mRNA Expression Profiles

NGS was used for examining the expression profiles of miRNAs and mRNAs. In brief, total RNA was extracted with Trizol^®^ Reagent (Invitrogen, Carlsbad, CA, USA), following the instruction manual. The purified RNAs were quantified at O.D._260_ with a ND-1000 spectrophotometer (Nanodrop Technology, Wilmington, DE, USA) and qualitatively assessed with Bioanalyzer 2100 and RNA 6000 Lab Chip kit (both from Agilent Technology, Santa Clara, CA, USA) to confirm the quality of extracted RNAs. Library preparation and sequencing were performed in Welgene Biotechnology Company (Taipei, Taiwan).

For transcriptome sequencing, the Agilent’s SureSelect Strand Specific RNA Library Preparation Kit was used to construct the libraries, followed by AMPure XP Beads size selection. Illumina’s sequencing-by-synthesis (SBS) technology was used for sequencing. Sequencing data (FASTQ files) were generated by Welgene’s pipeline based on Illumina’s base-calling program bcl2fastq v2.2.0. After adaptor clipping and sequence quality trimming with Trimmomatics (Ver. 0.36) [6], alignment of the qualified reads was performed using HISAT2 [7], a fast and sensitive alignment program for mapping NGS reads to genomes based on hierarchical graph FM index [7]. We excluded the genes with low expression levels (<0.03 fragment per kilobase of transcript per million mapped reads [FPKM]) in any group. The *p* values were calculated by Cuffdiff with non-grouped samples using the “blind mode”, in which all samples were treated as replicates of a single global “condition” and used to build a model for statistical test [8]. Genes with *p* value < 0.05 and >2-fold changes were considered significantly differentially expressed.

For small RNA sequencing, samples were prepared using Illumina sample preparation kit, following the instructions of the TruSeq Small RNA Sample Preparation Guide. The 3′ and 5′ adaptors were ligated to the RNA, and then reverse transcription and PCR amplification were performed. The cDNA constructs were size-fractionated and purified using a 6% polyacrylamide gel electrophoresis and the bands corresponding to the 18–40 nucleotide RNA fragments (140–155 nucleotide in length with both adapters) were extracted. After sequencing on an Illumina (San Diego, CA, USA) instrument (75 bp single-end reads), the data was processed with the Illumina software. After trimming and filtering out low-quality reads with Trimmomatics [6] and clipping the 3′ adapter sequence and discarding reads shorter than 18 nucleotides with miRDeep2 [9], the qualified reads were aligned to the human genome (version: GRCh38.p10) from University of California, Santa Cruz (UCSC). Because miRNAs usually map to few genomic locations, only reads mapped perfectly to the genome ≤5 times were taken. MiRDeep2 was used to estimate the expression levels of known miRNAs, as well as identifying novel miRNAs. The miRNAs with low expression levels (<1 normalized read per million (rpm)) in both groups were excluded. The miRNAs with >2-fold change are considered significantly differentially expressed.

### 2.3. Analyses Using miRNA Target Prediction Databases

TargetScan (http://www.targetscan.org) is an online database predicting the targets of miRNA by searching for the presence of conserved 8 mer, 7 mer, and 6mer sites matching the seed region of each miRNA [10]. The results of predictions are ranked by the predicted efficacy of targeting or by their probability of conserved targeting [11]. TargetScan is a valuable resource for investigating the role of miRNAs in gene-regulatory networks.

miRDB (http://mirdb.org) provides web-based miRNA-target prediction and functional annotations in five species, including human, mouse, rat, dog, and chicken [12]. In miRDB, all targets were predicted by MirTarget, which was developed by analyzing miRNA-target interactions from high-throughput sequencing experiments.

### 2.4. Analysis Using DAVID Database

DAVID (https://david.ncifcrf.gov/) is a powerful tool for functional classification of genes [13]. It integrates gene ontology and KEGG pathway. In DAVID database, a list of genes of interest can be uploaded and classified into clusters of related biological functions, signaling pathways, or diseases by calculating the similarity of global annotation profiles with an agglomeration algorithm method. An Expression Analysis Systematic Explorer (EASE) score is a modified Fisher’s exact *p* value in DAVID database which represents how specifically the genes are involved in a category. In this study, we selected EASE score = 0.1 as the default and defined pathways with a *p* value < 0.05 as significant.

### 2.5. Analysis Using IPA

IPA (Ingenuity systems, Redwood City, CA, USA) is a database software containing large databases with detailed and structured findings reviewed by experts, which was derived from thousands of biological, chemical and medical researches [14]. IPA enables rapid searching, analysis, integration, and recognition of data from gene and single nucleotide polymorphism (SNP) arrays, RNA and small RNA sequencing, proteomics and many other biological experiments. IPA can offer the information of related signaling pathways, upstream regulators, molecular interactions, disease process, and identify candidate biomarkers. In this study, we used IPA to assess the diseases and functions associated with the significantly dysregulated genes in corneal epithelial cells treated with 0.003% atropine. The disease and function with a *p* value < 0.05 were considered as significant.

## 3. Results

### 3.1. Differential Gene Expressions in Corneal Epithelial Cells Treated with Very Low Dose Atropine

**Figure 2:**
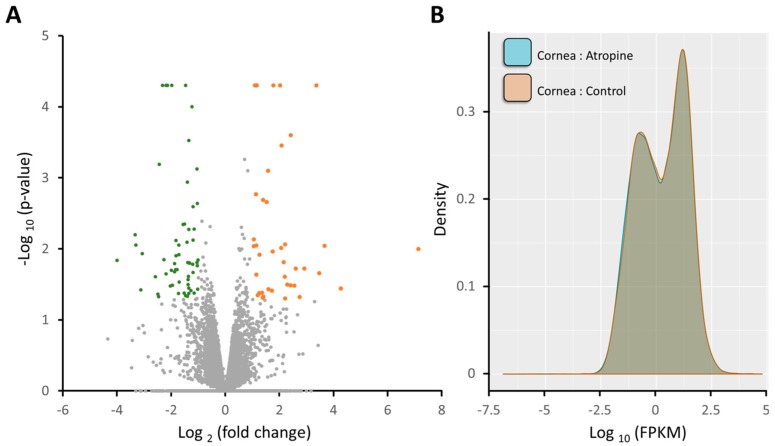
Overview of the gene expression profile in atropine-treated corneal epithelial cells. (**A**) The volcano plot of differential gene expression patterns of the normal corneal epithelial cells vs. 0.003% atropine-treated corneal epithelial cells. Significantly dysregulated genes in atropine-treated corneal epithelial cells (those with −log_10_(*p* value) > 1.3 and fold change > 2) were shown in green (downregulation) and orange (upregulation). (**B**) The density plot illustrates smoothed frequency distribution of the fragments per kilobase of transcript per million mapped reads (FPKM) among the normal corneal epithelial cells and atropine-treated corneal epithelial cells.

The volcano plots of differentially expressed genes between normal and atropine-treated corneal epithelial cells were shown in Figure 2A (0.003% Atropine vs. control) with significantly dysregulated genes (fold change > 2 and *p* value < 0.05) indicated in colored plots. A total of 40 significantly upregulated genes and 60 significantly downregulated genes in atropine-treated corneal epithelial cells were identified. The gene expression profiles in density plot of normal and atropine-treated corneal epithelial cells were shown in Figure 2B. These significantly dysregulated genes were listed in Table 1.

### 3.2. Function and Pathway Analysis of Dysregulated Genes in Atropine-Treated Corneal Epithelial Cells

**Figure 3:**
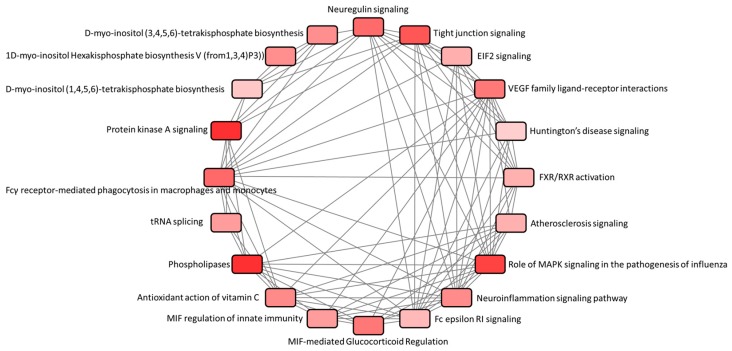
Canonical pathway analysis of the significantly dysregulated genes in atropine-treated corneal epithelial cells. Using Ingenuity^®^ Pathway Analysis (IPA), the associated canonical pathways of 100 significantly dysregulated genes in atropine-treated corneal epithelial cells were analyzed. The interaction network between these canonical pathways were generated.

We analyzed these 100 dysregulated genes in the IPA. The interactions between different pathways associated with these 100 dysregulated genes were shown in Figure 3. Among the identified pathways, protein kinase A (PKA) signaling and neuroinflammation signaling pathway were the two pathways with positive predictive z-scores, as listed in Table 2, which indicated activated pathways among the dysregulated genes. In the disease and function analysis of IPA, in atropine-treated corneal epithelial cells, 2 functions associated with negative z-scores were listed in Table 3, including cell death and apoptosis, which indicated suppressed functions.

Furthermore, the 100 dysregulated genes were uploaded into DAVID database for KEGG pathway analysis. The results identified six significantly dysregulated pathways that were involved in atropine-treated corneal epithelial cells (Table 4), including glucagon signaling pathway, estrogen signaling pathway, Ras signaling pathway, insulin signaling pathway, arachidonic acid metabolism, and hepatitis B.

### 3.3. Potential Dysregulated miRNA-mRNA Interactions in Atropine-Treated Corneal Epithelial Cells

Using NGS, 678 significantly dysregulated miRNAs (>1-fold change, including 336 upregulated and 341 down regulated miRNAs) were identified. We predicted the potential targets of these miRNAs using miRDB database and those with miRDB score ≥ 97.0 were selected. Matching to the mRNAs with at least two-fold change between normal and atropine-treated corneal epithelial cells, the results yielded 12 potential miRNA-mRNA interactions (including 4 upregulated mRNAs with 3 potential miRNA regulations and 8 downregulated mRNAs with 4 potential miRNA regulations). Further validation using TargetScan database revealed that only hsa-miR-651-3p-*EPHA7*, hsa-miR-3148-*TMEM108*, and hsa-miR-874-5p-*TBX6* were validated in both TargetScan and miRDB databases, as listed in Table 5.

## 4. Discussion

**Figure 4:**
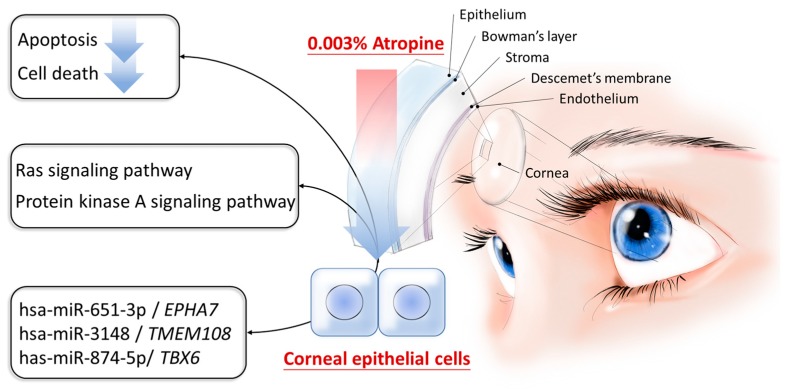
Schematic summary of molecular mechanisms potentially involved in atropine-treated corneal epithelial cells.

In the current study, we investigated the role of atropine in the alteration of gene expression profiles of corneal epithelial cells using NGS and bioinformatics tools. Firstly, 100 significantly dysregulated genes were identified. Among them, 40 genes were significantly upregulated and 60 genes were significantly downregulated. To determine biological functions and signaling pathways potentially involved in atropine-treated corneal epithelial cells, we investigated these 100 dysregulated genes using different bioinformatics databases. The dysregulated genes were enriched in PKA signaling, neuroinflammation signaling, Ras signaling and metabolism-related pathways, while functions related to cell death and apoptosis were predicted to be suppressed in atropine-treated corneal epithelial cells. Atropine has been reported to exert dose-dependent cytotoxic effect to human corneal epithelial cells and corneal endothelial cells at concentrations above 0.03% [2,15]. In the current study, very low dose atropine (0.003%) was treated to corneal epithelial cells. Through NGS and bioinformatics analysis, the results suggested that very low dose atropine treatment to the corneal epithelial cells may suppress cell death and apoptosis through Ras signaling pathway and PKA signaling pathways. The schematic summary is illustrated in Figure 4.

Atropine at different concentrations have been widely used as topical eye drops in slowing the progression of myopia in children, particularly in the Asian area [1]. From the review and meta-analysis, the current lowest effective concentration of atropine in myopia control is reported to be 0.01%, controlling the axial growth with minimal side effects [16,17]. However, the studies focused on children with myopia, and the effective dose of atropine in myopia control as one of the treatment modalities. The cytotoxic effect of atropine to corneal epithelial cells at concentrations above 0.03% has been reported [2]. Based on the current evidence and the uncertainty of potential side effects with long term use of atropine [18], we are interested in whether very low concentration of atropine exerts preventive role in myopia prevention in children. Since cornea is the tissue in direct contact to topical eye drops, cytotoxicity to corneal tissues should be a major concern. Thus, the current study focused on investigating the potential effects of very low dose atropine (0.003%) on corneal epithelial cells, using NGS and bioinformatics approaches. The current results suggested the potential involvement of suppressed cell death and apoptosis in very low dose atropine treated corneal epithelial cells. Therefore, we postulated that the preventive use of very low dose atropine may be of minimal side effects. However, the potential clinical effect of very low dose atropine in preservation of chamber length and myopia prevention needs future investigation.

In the study of spinal cord injury, inhibition of EPHA7 expression was proved to reduce apoptosis and accelerate hindlimb locomotor recovery, and EphA7 receptors were demonstrated as putative regulators of apoptosis in the acute phase after spinal cord injury [19]. In laryngeal squamous cell carcinoma, upregulated EphA7 was observed in human laryngeal squamous cell carcinoma samples, while down-regulation of EphA7 effectively suppressed cell growth and promoted cell apoptosis [20]. These studies suggested that EphA7 plays an important role in the modulation of cell growth and apoptosis. In our NGS results, downregulated *EPHA7* was observed in atropine-treated corneal epithelial cells. We postulated that downregulated *EPHA7* might be associated with suppression of apoptosis in corneal epithelial cells treated with 0.003% atropine.

*TMEM108* is a risk gene of psychiatric diseases. High expression of TMEM108 was observed in the dentate gyrus and CA3 of the hippocampus. In the study of TMEM108 mutant mice, TMEM108 is involved in adult neurogenesis, and ablation of TMEM108 was demonstrated to decrease the proliferation of dentate gyrus neuronal progenitor cells [21]. TMEM108 is reported to express specifically in the nervous system, and is critical in neural development and function [22]. However, the role of TMEM108 in corneal cells remain unknown. Research suggested the interaction between keratitis and corneal epithelial dendritic cells [23]. In our NGS data, the expression of *TMEM108* is upregulated. We may propose that the expression of *TEME108* will change in corneal nerves and the innervated corneal tissue after atropine treatment, and further investigation is necessary to determine the expression level of *TMEM108* in corneal tissue.

*TBX6* belongs to the T-box family of transcription factor genes and has mutant alleles. The loss-of-function mutation of the mouse Tbx6 gene will lead to a profound disturbance in somite development [24]. In the study of primary cultured postnatal rat cardiomyocytes, TBX6 could activate cell cycle and induce proliferation of cardiomyocytes, but not the cardiac fibroblasts. Furthermore, the overexpression of TBX6 could upregulate multiple cell cycle activators [25]. In our NGS data, the expression of *TBX6* is upregulated. We postulated that upregulated *TBX6* in very low dose atropine treatment may possibly activate cell cycle and induce cell proliferation, instead of exerting cytotoxic apoptotic effect in higher doses of atropine previously reported [2]. This may provide evidence of protective effect of atropine to corneal epithelial cells in very low concentrations.

## 5. Conclusions

In summary, we identified 100 significantly dysregulated genes in 0.003% atropine-treated corneal epithelial cells. These genes were involved in suppressed cell death and apoptosis. The possible pathways involved in atropine-treated corneal epithelial cells might be Ras signaling pathway and PKA signaling. We also identified 3 pairs of miRNA-mRNA interactions, including hsa-miR-651-3p-*EPHA7*, hsa-miR-3148-*TMEM108*, and hsa-miR-874-5p-*TBX6.* These findings may contribute important new insights into novel targets and effects in corneal tissue while elucidating the effect of very low dose atropine in the prevention of myopia.

## Figures and Tables

**Table 1:** Significantly differentially expressed genes in atropine-treated corneal epithelial cells.

**Upregulated Genes**
*RPL34*, *ACACA*, *CUTA*, *AP2A2*, *ITPK1*, *IFI27*, *ERMARD*, *ZNF578*, *ZNF487*, *TBCE*, *HHAT*, *CBS*, *PCOLCE2*, *CTIF*, *UBR7*, *NEK10*, *KCNJ5*, *FAM156A*, *NAPEPLD*, *IRF7*, *PTEN*, *C16orf45*, *SMN2*, *CFB*, *PYGM*, *PLA2G3*, *PRSS27*, *RPS18*, *GGT5*, *GBA*, *PYCR3*, *CALML6*, *KIF6*, *NRG4*, *ARHGAP15*, *ART3*, *JAML*, *TIE1*, *ARHGAP11B*, *LGR5*
**Downregulated Genes**
*SQSTM1*, *SYNRG*, *PTPRK*, *COG5*, *SLC25A26*, *PSMC4*, *CEP170*, *TC2N*, *FOPNL*, *NSF*, *AKT3*, *SLX1B*, *FAM120B*, *STAT4*, *PIF1*, *IQANK1*, *PON1*, *FRG1*, *LEKR1*, *AC145212.1*, *STK33*, *CRIP1*, *PLA2G4C*, *LIMS4*, *CDC20*, *HORMAD1*, *GGN*, *FOXA1*, *CIT*, *APBB1*, *BUB1B-PAK6*, *TBX3*, *SLAIN1*, *UGT2B10*, *IFI27L1*, *CCHCR1*, *FAM111B*, *DHRS11*, *SMN1*, *POLQ*, *RPL7A*, *ZNF100*, *PQLC2L*, *RASA3*, *OXER1*, *FOLH1*, *PLEKHG4*, *BEGAIN*, *RNF150*, *NCALD*, *OCLN*, *MCM10*, *CHST6*, *DUSP8*, *COL14A1*, *PDE9A*, *ZKSCAN7*, *HSPA6*, *FSTL4*, *PRLR*

**Table 2:** Potentially activated canonical pathways in atropine-treated corneal epithelial cells.

Canonical Pathways	−log_10_(*p*-Value)	Ratio	z-Score	Molecules
Protein Kinase A Signaling	2.69	0.0182	0.378	*NAPEPLD*, *HHAT*, *PYGM*, *DUSP8*, *PTPRK*, *PDE9A*, *PTEN*
Neuroinflammation Signaling Pathway	1.87	0.0165	0.447	*IRF7*, *KCNJ5*, *PLA2G4C*, *AKT3*, *PLA2G3*

**Table 3:** Diseases and functions involved in atropine-treated corneal epithelial cells.

Categories	Functions	Diseases or Functions Annotation	*p*-Value	Activation z-Score	Molecules
Cell death and survival	Cell death	Cell death of colorectal cancer cell lines	0.0116	−0.468	*SQSTM1*, *PTEN*, *AP2A2*, *RPL34*, *CDC20*, *LGR5*
Cell death and survival	Apoptosis	Apoptosis	0.0252	−0.103	*SQSTM1*, *IFI27L1*, *PTEN*, *AKT3*, *MCM10*, *OCLN*, *IFI27*, *CRIP1*, *CDC20*, *CCHCR1*, *ITPK1*, *FOXA1*, *AP2A2*, *CIT*, *TBX3*, *APBB1*, *ACACA*, *SMN1/SMN2*, *LGR5*

**Table 4:** Possible pathways involved in atropine-treated corneal epithelial cells analyzed by DAVID.

Pathway	*p*-Value	Genes
Glucagon signaling pathway	0.018	*PYGM*, *ACACA*, *CALML6*, *AKT3*
Estrogen signaling pathway	0.018	*KCNJ5*, *HSPA6*, *CALML6*, *AKT3*
Ras signaling pathway	0.038	*CALML6*, *PLA2G4C*, *PLA2G3*, *RASA3*, *AKT3*
Insulin signaling pathway	0.043	*PYGM*, *ACACA*, *CALML6*, *AKT3*
Arachidonic acid metabolism	0.046	*GGT5*, *PLA2G4C*, *PLA2G3*
Hepatitis B	0.048	*STAT4*, *IRF7*, *PTEN*, *AKT3*

**Table 5:** Potential miRNA-mRNA interactions involved in atropine-treated corneal epithelial cells.

	Predicted Consequential Pairing of Target Region (Top) and miRNA (Bottom)	Context++ Score	Context++ Score Percentile	Weighted Context++ Score	miRDB
Position 1034–1040 of *EPHA7* 3′ UTR **miR-651-3p**	5′ … UGCAGACAUCCUCCAUUUCCUUU… | | | | | | | 3′ GAAAAUCCUAUGUGAAAGGAAA	−0.02	37	0.00	97
Position 3122–3128 of *EPHA7* 3′ UTR **miR-651-3p**	5′ …AUAGUAUUCUUCCUGUUUCCUUU… | | | | | | | 3′ GAAAAUCCUAUGUGAAAGGAAA	−0.02	37	0.00	97
Position 1932-1938 of *TMEM108* 3′ UTR **miR-3148**	5′ …CACUUUAGGCCACAG - - UUUUUCCU… | | | | | | | | | | 3′ UUCGUGUGUGGUCAAAAAAGGU	−0.02	38	−0.02	99
Position 204-210 of *TBX6* 3′ UTR **miR-874-5p**	5′ …UGCUGGGAGUCCAGCUGGGGCCG… | | | | | | | 3′ AGAAUGGGACCACGCACCCCGGC	−0.10	57	−0.10	94
Position 269-276 of *TBX6* 3′ UTR **miR-874-5p**	5′ …CUCUGCCUGGCCAAAUGGGGCCA… | | | | | | | | | | | | 3′ AGAAUGGGACCACGC - ACCCCGGC	−0.47	98	−0.47	94
